# Toward Biomimetic Scaffolds for Tissue Engineering: 3D Printing Techniques in Regenerative Medicine

**DOI:** 10.3389/fbioe.2020.586406

**Published:** 2020-11-04

**Authors:** Justin J. Chung, Heejung Im, Soo Hyun Kim, Jong Woong Park, Youngmee Jung

**Affiliations:** ^1^Center for Biomaterials, Korea Institute of Science and Technology, Seoul, South Korea; ^2^KU-KIST Graduate School of Converging Science and Technology, Korea University, Seoul, South Korea; ^3^Department of Orthopedic Surgery, Korea University Anam Hospital, Seoul, South Korea; ^4^School of Electrical and Electronic Engineering, Yonsei University, Seoul, South Korea

**Keywords:** 3D printing, tissue engineering, bioink, scaffold, regenerative medicine

## Abstract

Three-dimensional (3D) printing technology allows fabricating complex and precise structures by stacking materials layer by layer. The fabrication method has a strong potential in the regenerative medicine field to produce customizable and defect-fillable scaffolds for tissue regeneration. Plus, biocompatible materials, bioactive molecules, and cells can be printed together or separately to enhance scaffolds, which can save patients who suffer from shortage of transplantable organs. There are various 3D printing techniques that depend on the types of materials, or inks, used. Here, different types of organs (bone, cartilage, heart valve, liver, and skin) that are aided by 3D printed scaffolds and printing methods that are applied in the biomedical fields are reviewed.

## Introduction

Three-dimensional (3D) printing, also known as additive manufacturing, is a method that can fabricate objects with complex structures by depositing materials, i.e., metals, polymers, and ceramics, layer by layer (He et al., 2014; Hung et al., 2014). A 3D object can be produced through 3D scanning technology, such as computed tomography (CT) and computer-aided design (CAD) software (Hollister, 2005; Melchels et al., 2010). After image file of an object is acquired; it is converted to an STL file format that can be sliced into layers to create a 3D model (Melchels et al., 2010; Zorlutuna et al., 2012). Charles W. Hull, the president of 3D SYSTEMS, invented the first 3D printer, which was based on a stereolithography apparatus (SLA) technique (Melchels et al., 2010; Murphy and Atala, 2014; Schubert et al., 2014). The SLA printing method obtained issued a patent in 1986 (Gross et al., 2014). In 1990, a fused deposition modeling (FDM)-type printer was developed by Scott Crump, chairman of STRATASYS (Rengier et al., 2010; Gross et al., 2014). These 3D printing techniques sent shockwaves throughout multiple industries, such as automotive, aerospace, architecture, fashion, as well as bio-medicine (Duoss et al., 2014; Gross et al., 2014), since complex 3D structures can be precisely controlled and easily produced compared to subtractive methods (He et al., 2014; Choi and Kim, 2015).

In the medical fields, 3D printing technology is a promising tool for personalized treatments (Choi and Kim, 2015; Jakus et al., 2015; Kim et al., 2016). 3D models of a patient’s damaged organ can be produced to serve as a visual aid for the surgeons and to help the patient to understand his or her conditions (Melchels et al., 2010; Gross et al., 2014). Additionally, 3D printing techniques are applied to produce scaffolds and implants for regenerative medicine (He et al., 2014; Torres-Rendon et al., 2015; Kim et al., 2018, Lee et al., 2019). For example, Mankovich et al. (1994) used additive manufacturing to fabricate calvarial bone grafts, and Morrison et al. (2015) designed 3D printed scaffolds to treat tracheobronchomalacia. There are various 3D printing techniques that are classified by the types of materials and printing methods that are used to create an object (Table 1; Sachlos and Czernuszka, 2003; Childers et al., 2015). FDM is the most common 3D printing method. Thermoplastic filaments are melted by a heating block, and then a nozzle head directs the extrusion of the melted filaments to deposit thin layers (Azari and Nikzad, 2009; Gross et al., 2014). One of the advantages of FDM is that there are wide ranges of biodegradable and biocompatible materials, or filaments, which can be printed. Additionally, toxic organic solvents are not required to dissolve the polymeric filaments for printing (Yang et al., 2002; Leong et al., 2003). For example, Hutmacher et al. (2001) fabricated poly(ε-caprolactone) (PCL) scaffolds with a honeycomb-like porous structure using an FDM-type printer. Fibroblasts were able to proliferate and differentiate on the scaffold. However, the high temperature applied during the melt-extrusion stage can change the inherent material properties, and high-resolution printing is challenging (Yang et al., 2002). SLA type is based on solidification of liquid resin through photo-crosslinking (Melchels et al., 2010). A stage, or a base plate, for an object is immersed in liquid resin, and then the laser beam is applied to cure the resin on the stage. After the first layer is produced, the stage moves downward and the second layer is cured to deposit on the first layer (Gross et al., 2014). A selective laser sintering (SLS) printer follows a similar process to the SLA type, but the high-powered laser is applied to sinter solid powders (Leong et al., 2003). SLA-type printers can produce objects with high resolution and design more precise structures compared to the FDM technique. However, they are limited to photo-polymerizing resins, and the resins are often toxic for biomedical applications. The SLS method does not require liquid resins or toxic organic solvents to dissolve polymers, yet the sintering process can damage materials that are biodegradable (Yang et al., 2002; Williams et al., 2005). 3D plotting is an extrusion-based technology, which expels materials from a chamber by pneumatics (Sachlos and Czernuszka, 2003). Typically, plotting pastes and viscose materials are used as printing inks (Luo et al., 2013), which are either directly printed or melted in a feeding channel before they are extruded by a pneumatic pump (Ragaert et al., 2010). Due to the mild printing conditions, various soft materials (i.e., hydrogels, biocompatible polymers, and cell spheroids) can be printed with the 3D plotting method, and it is also referred to as bioprinting when cells are printed with hydrogel inks (Murphy and Atala, 2014). Haberstroh et al. (2010) were able to fabricate 3D plotted cell-seeded scaffolds of poly(L-lactide-co-glycolide) (PLGA), tricalcium phosphate (TCP)/collagen, and TCP/collagen/chitosan successfully for bone regeneration. The bioactivity of scaffolds and bone formation in calvarial defect model were evaluated. Lee et al. (2019) developed a new type of 3D bioprinting method, which allows printing a more precise and complex organ structure using hydrogel inks. The collagen ink was printed within a thermo-reversible support bath of gelatin microparticles to reproduce patient-specific cardiac ventricles. Inkjet printing is also widely used in regenerative medicine. It is a droplet-based extrusion printing technique, where droplets from the supplied fluid are deposited layer by layer, or patterned to desired shapes with biomolecules (Xu et al., 2013). Inkjet printing methods are cost-effective and applied in various fields from drug screening to tissue engineering (Boland et al., 2006); however, it is challenging to print viscous materials and cells (Koch et al., 2010). The laminated object manufacturing (LOM) process builds polymeric and metallic layers that are sequentially fed from a roller (Park et al., 2000). Laminates are cut with CO*2* laser, and then layers are bonded by a heated roller. This rapid prototyping process can fabricate large objects with low cost (Murr(ed.)., 2015); however, it is challenging to make small and precise structures (Yang et al., 2002).

**TABLE 1:** Different types of 3D printing techniques, and their pros and cons.

**Types**	**Advantage**	**Limitations**	**References**
FDM	Thermoplastic polymers are extruded without toxic organic solvents	Melting process can affect inherent material properties	Cornejo et al., 2000; Hutmacher et al., 2001; Yang et al., 2002; Zein et al., 2002; Leong et al., 2003; Woodfield et al., 2004; Azari and Nikzad, 2009; Gross et al., 2014; Hung et al., 2014, 2016; Kwon et al., 2015
SLA	High-resolution objects can be printed with complex structures	Printable materials are limited to liquid resins, which can be toxic	Rimell and Marquis, 2000; Leong et al., 2003; Tsang and Bhatia, 2004; Jiankang et al., 2009; Melchels et al., 2010; Gross et al., 2014; Melchiorri et al., 2016
SLS	Powdered materials are sintered through a similar process as SLA. No liquid resins are needed	Sintering can modify material properties	Yang et al., 2002; Seitz et al., 2005; Williams et al., 2005; Gbureck et al., 2007; Khalyfa et al., 2007; Morrison et al., 2015; Pei et al., 2017; Zhang et al., 2019
3D plotting	Bioceramics can be printed in mild conditions. 3D cell printing is possible by seeding cells into hydrogels	Post-sintering or curing is necessary. Constrained by temperature and complex multi-layer fabrication is challenging for bioprinting	Sachlos and Czernuszka, 2003; Wang et al., 2006; Lee et al., 2009, 2014b, 2019; Haberstroh et al., 2010; Ragaert et al., 2010; Fu et al., 2011; Wu et al., 2011; Hockaday et al., 2012; Bose et al., 2013; Gao et al., 2013; Luo et al., 2013; Mannoor et al., 2013; Duan et al., 2014; Inzana et al., 2014; Murphy and Atala, 2014; Markstedt et al., 2015; Kim et al., 2018; Nommeots-Nomm et al., 2018; Tallia et al., 2018; Li et al., 2020; Shi et al., 2020
Inkjet	Precise and controlled placement printing for small volume biological materials	Difficult to print viscose materials/cells. Large volume construct fabrication is challenging	Boland et al., 2006; Cui and Boland, 2009; Koch et al., 2010; Xu et al., 2013
LOM	Ideal for fabricating large 3D objects	Lamination coatings can be toxic and vulnerable when fabricating small constructs	Park et al., 2000; Yang et al., 2002; Murr(ed.)., 2015

**FIGURE 1:**
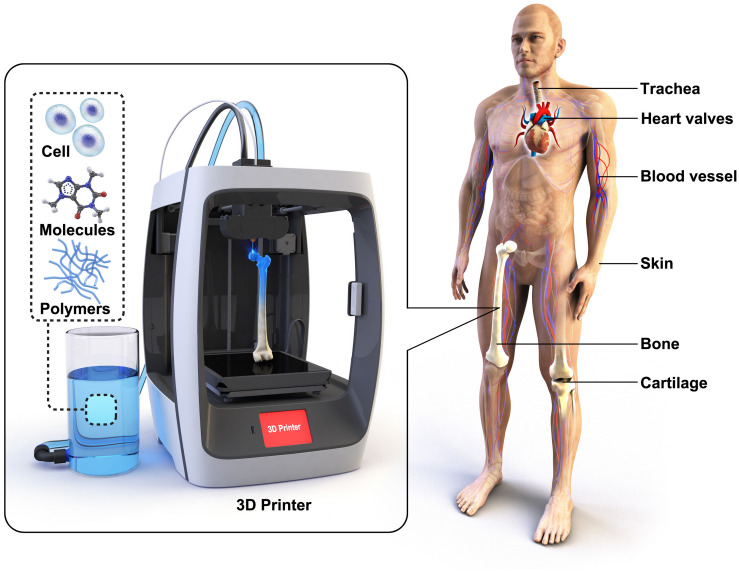
Various scaffolds for tissues and organs have been 3D printed in the regenerative medicine field. 3D scaffolds are typically made of biocompatible polymers. Cells and bioactive molecules are often incorporated with the scaffolds to enhance bioactive properties.

Every year, millions of patients are waiting organ donors and suffer from long transplant waiting lists. Tissue engineering has a potential not only to solve the current complications in organ shortage but also to improve the current level of the biomedical technology (Khademhosseini et al., 2006; Slaughter et al., 2009). Vacanti et al. (1988) established seminal work in the field of tissue engineering in the 1980s, and it is still one of the most researched fields. Conventionally, most scaffolds for tissue engineering were fabricated through a “top-down” approach, where scaffolds are designed with biocompatible polymeric materials in porous structures to biomimic the host tissue. However, for cell attachment and proliferation, the scaffolds were coated with bioactive substances, or surface modification was necessary. In contrast, the “bottom-up” approach aims to encapsulate cells in hydrogels to allow self-assembly of cell aggregation and 3D print cells directly in the form of a scaffold (Figure 1). An ideal scaffold has to possess a surface that is suitable for cell attachment and 3D inter-connected porous structures for extracellular matrix (ECM) formation and vascularization. 3D printing allows fabricating scaffolds with more controlled and precise structures (Derby, 2012; Do et al., 2015) compared to electro-spinning (Poologasundarampillai et al., 2011), foaming (Mahony et al., 2010), freeze-drying (Connell et al., 2014), and salt-leaching (Woodard et al., 2017) techniques. Here, we review 3D printing technologies for regenerative medicine, from 3D printed polymeric scaffolds to bio-artificial tissues, and promising outlooks for advanced treatments through 3D printing.

## 3D Printed Scaffolds for Tissue Engineering

There have been numerous types of 3D printing techniques used and developed by researchers in the field of tissue engineering. In this section, 3D printing techniques and 3D printed biomaterials are categorized into subsections by tissues and organs that they were designed to aid. They are also summarized in Table 2.

**TABLE 2:** Summary of materials, cell types, and molecules used for 3D printed scaffolds for tissue engineering.

**Organs**	**Materials**	**Cell type/Molecules**	**Printer type**	**References**
Bone	PCL	BMP-2	SLS	Williams et al., 2005
	PLGA, TCP, Collagen, Chitosan	–	FDM and 3D plotting	Haberstroh et al., 2010
	PLGC		FDM	Kwon et al., 2015
	TTCP, β-TCP, Calcium sulfate	–	SLS	Khalyfa et al., 2007
	TCP	–	SLS	Gbureck et al., 2007; Zhang et al., 2019
	HA	–	SLS	Seitz et al., 2005; Pei et al., 2017; Zhang et al., 2019
	Calcium phosphate, Type I collagen	–	3D plotting	Inzana et al., 2014
	PCL, Type I collagen, Alginate, Gelatin	DPSCs/VEGF, BMP-2	FDM and 3D plotting	Park et al., 2015
	Bioactive glasses	–	3D plotting	Fu et al., 2011; Nommeots-Nomm et al., 2018; Shi et al., 2020
	Bioglass–gelatin hybrid	–	3D plotting	Gao et al., 2013
Cartilage	PU, PEO	–	FDM	Hung et al., 2014, 2016
	PEGT/PBT block copolymer	–	FDM	Woodfield et al., 2004
	Cellulose, Alginate	Human nasoseptal chondrocytes	3D plotting	Markstedt et al., 2015
	PCL, PEG, Alginate	Chondrocytes, Adipocytes	FDM and 3D plotting	Lee et al., 2014a
	Silicon, Alginate, Silver nanoparticle	Calf articular chondrocyte	3D plotting	Mannoor et al., 2013
	Silica-Poly(tetrahydrofuran)-PCL-hybrid	–	3D plotting	Tallia et al., 2018; Li et al., 2020
Heart valve	PEG-DA, Alginate	PAVIC	3D plotting	Hockaday et al., 2012
	Me-HA, Me-Gel	HAVIC	3D plotting	Duan et al., 2014
	Collagen	Human embryonic stem cell-derived cardiomyocytes	3D plotting	Lee et al., 2019
Blood vessel	Pluronic F127-DA	–	3D plotting	Wu et al., 2011
	Poly(propylene fumarate)	–	SLA	Melchiorri et al., 2016
	Type I collagen, Gelatin	HUVECs, ECs	3D plotting	Lee et al., 2014b
	Fibrinogen, Thrombin, CaCl_2_	HMVECs	Inkjet	Cui and Boland, 2009
Trachea	PCL, Hydroxyapatite	–	SLS	Morrison et al., 2015
	PCL	–	FDM	Chang et al., 2014
	Silk fibroin	Chondrocytes	3D plotting	Kim et al., 2018
Liver	Gelatin	Hepatocytes	3D plotting	Wang et al., 2006
	PDMS	–	SLA	Jiankang et al., 2009
	PCL, Collagen	Hepatocytes, HUVECs, HLFs	FDM and 3D plotting	Lee et al., 2016
Skin	Type I collagen	hDFB, hEKC	3D plotting	Lee et al., 2009
	Collagen	NIH-3T3, HaCaT	Inkjet	Koch et al., 2012

### Bone

The bone regeneration process involves migration and recruitment of osteoprogenitor cells to a defect region, which will then differentiate to osteoblasts to form bone minerals or hydroxyapatite (HA). There are various methods to enhance bioactive properties to bone tissue engineering scaffolds, such as incorporating growth factors and gene/drug deliveries. An ideal bone scaffold should be made with biocompatible materials that act as a temporary template to withstand mechanical forces in the defect site until the host tissue is fully recovered. Specifically, biodegradation rate should be similar to the duration of bone formation process, and inter-connected porous structure for vascularization is essential for the scaffold design (Bose et al., 2012; Jones, 2013).

**FIGURE 2:**
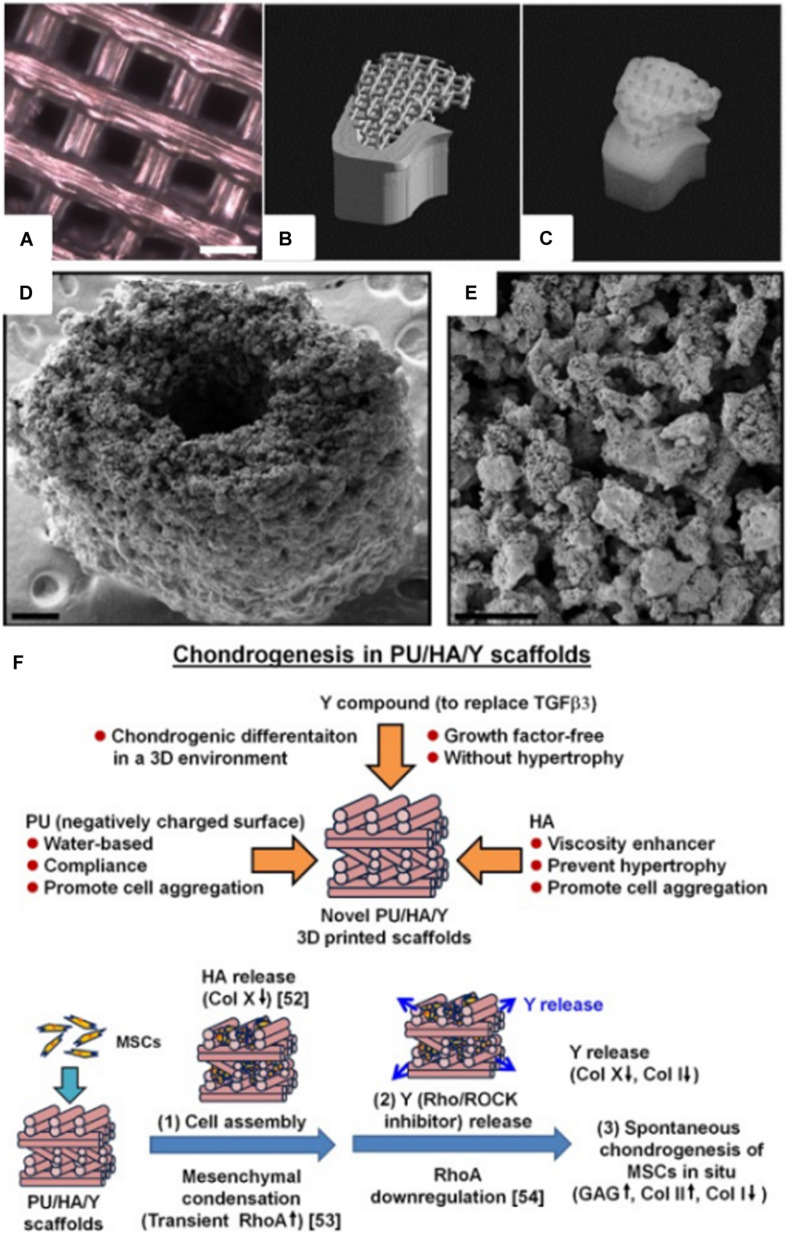
**(A)** Optical microscope image of a 3D printed poly(l-lactide-co-ε-caprolactone) scaffold for adipose tissue engineering (courtesy of YJ, scale bar = 2 mm). **(B)** STL image of a pig condyle scaffold. **(C)** Front view of the 3D printed PCL scaffold (Williams et al., 2005). **(D)** SEM image of a 3D printed murine-sized scaffold for femoral mid-diaphysis regeneration (scale bar = 250 μm). **(E)** Micro-porosity of the calcium phosphate-collage composite scaffold with pore sizes of 20–50 μm (scale bar = 100 μm) (Inzana et al., 2014). **(F)** 3D printed PU/HA-based scaffold design, and possible mechanism of spontaneous chondrogenesis *in situ* (Hung et al., 2016) (Reproduced with permission from Williams et al., 2005; Inzana et al., 2014; Hung et al., 2016).

Synthetic biodegradable polymers, such as poly(caprolactone) (PCL), poly(glycolic acid) (PGA), poly(lactic acid) (PLA), and their copolymers, have received high attention in the biomedical field since ester linkages can be degraded by hydrolysis and their by-products are non-toxic. These biodegradable polymers have also been 3D printed to produce scaffolds for tissue engineering (Figure 2A). Williams et al. (2005) fabricated PCL scaffold with similar mechanical properties to that of human trabecular bone using the SLS printing technique. Bone morphogenetic protein-7 (BMP-7) was seeded to enhance the bioactivity of the scaffold, and subcutaneous implantation has shown bone formation within 4 weeks. Additionally, the scaffold was fabricated to replicate a CT-scanned minipig condyle structure, which showed a possibility of producing patient-specific scaffolds (Figures 2B,C). Kwon et al. (2015) synthesized PCL containing PLGC [methoxy poly(ethylene glycol)-co-L-lactide-co-glycolide-co-ε-caprolactone] copolymer scaffold through the FDM process. Human dental pulp stem cell (hDPSC)-loaded PLGC scaffold instigated bone regeneration, and the degradation rate of the scaffold was comparable to the bone formation rate.

Calcium phosphate-based ceramics have similar compositions to bone mineral; therefore, they have been widely used as bone substitutes. Since bioceramics are in powder form, the SLS method is often used to produce grid-like scaffold structures (Seitz et al., 2005; Gbureck et al., 2007; Khalyfa et al., 2007). 3D plotting is also practiced with a post-sintering process when binders and sacrificial polymers are mixed with bioceramics to produce printable inks (Pei et al., 2017; Zhang et al., 2019). Although bioactive glasses are not commercially successful as bioceramics, they are known to be more bioactive, and 45S5 composition (Bioglass^®^) was the first artificial material that formed a chemical bond to bone (Hench, 2006; Jones, 2013; Cai et al., 2018). Various compositions of bioactive glasses were also 3D printed to biomimic the porous structure of bone (Fu et al., 2011; Nommeots-Nomm et al., 2018; Shi et al., 2020). However, inorganic scaffolds (i.e., bioceramics and bioactive glasses) are too brittle for repairing defect sites that are exposed to constant loading. Flexibility and toughness of bioactive glasses can be enhanced by introducing flexible polymers to the silica network with covalent bonding, termed inorganic–organic hybrids. This is possible since glasses can be fabricated through a sol-gel process that prevents oxidization of polymers (Sanchez et al., 2005; Jones et al., 2006; Valliant and Jones, 2011; Chung et al., 2017). Gao et al. (2013) were able to 3D print a gelatin–bioactive glass hybrid to a grid-like structure. MC3T3-E1 osteoblast precursor cells were able to adhere and proliferate on the printed hybrid scaffold.

Bone is a nanocomposite composed of HA (50–70%) and organic matrix (20–40%), which is primarily composed of type I collagen (Clarke, 2008; Jones, 2013). Collagen is widely used as biomaterials for tissue engineering in skin (Powell et al., 2008), bone (Rodrigues et al., 2003), tendon (Young et al., 1998), and blood vessel (Zorlutuna et al., 2008) applications due to its tough mechanical properties and biocompatibility. Inzana et al. (2014) 3D printed collagen and calcium phosphate composite for bone regeneration. The scaffold was implanted in a murine femur with critical defect size, and osteoconductivity was confirmed (Figures 2D,E). 3D bioprinting technique is one of the most recent methods of printing biomaterials; it renders 3D tissue constructs with cells embedded in hydrogels (Mironov et al., 2009; Kang et al., 2013; Levato et al., 2014). Park et al. (2015) were able to demonstrate the multi-head bioprinting method. Vascular endothelial growth factor (VEGF) and BMP-2 were loaded to various blends of hydrogels with hDPSC. The hydrogels were 3D plotted to a PCL scaffold framework, and this fabrication method was shown to produce large volume scaffolds, which is one of the major limitations in the tissue engineering field.

### Cartilage

The ECM of cartilage is composed of type II collagen and glycosaminoglycan (GAG), which allows to regulate expression of chondrocyte phenotype and instigate chondrogenesis (Suh and Matthew, 2000). In contrast to bone, cartilage has limited ability to naturally heal itself, since avascular structure inhibits nutrients and progenitor cells to migrate toward the defect region. Articular cartilage covers end of bones in synovial joints, which allows the bones to glide over each other; therefore, it should withstand load-bearing forces while providing low-friction surfaces. Osteoarthritis and high-impact injuries can cause articular cartilage defects, and it is one of the most challenging tissues to repair (Temenoff and Mikos, 2000; Huey et al., 2012).

Poly(ethylene glycol) terephthalate/poly(butylene terephthalate) (PEGT/PBT) block copolymer was 3D printed through the FDM technique to fabricate grid-like structured scaffold. The scaffold was seeded with bovine articular chondrocytes (bACs), which developed cartilage-like tissue *in vivo* while having mechanical properties similar to the native articular cartilage (Woodfield et al., 2004). Hung et al. (2014, 2016) were able to 3D print cartilage scaffolds with water as a printing ink solvent, which allowed incorporation of biomolecules, i.e., growth factors, with higher biocompatibility compared to inks that require organic solvents. Polyurethane (PU) particles, hyaluronic acid (HA), and TGFβ3 containing ink were 3D printed through a customized low-temperature FDM-type printer. Then, the scaffold was seeded with mesenchymal stem cells (MSCs) to improve cartilage regeneration *in vivo* (Figure 2F). Markstedt et al. (2015) developed a nanocellulose-alginate scaffold using the 3D bioprinting technique. The rheological properties of the composite bioink, which required low pressure to extrude at room temperature, allowed the production of precise 3D grid, disc, human ear, and sheep meniscus constructs. The cytotoxicity and live/dead cell-imaging assay confirmed that the scaffold was suitable for cartilage regeneration.

Other researchers have also reported 3D printing of ear-shaped structures for cartilage regeneration. Lee et al. (2014a) fabricated a human ear scaffold by printing both articular cartilage and fat tissue. Poly(ethylene glycol) (PEG) was used as a sacrificial layer since it is soluble in aqueous solutions, and PCL was printed as a main framework of the scaffold. Alginate hydrogel was used as a bioink to print chondrocyte and adipocyte. It was printed along with PCL to fabricate an ear scaffold with two distinct regions, which included a main ear part (chondrocyte) and an earlobe area (adipocyte). Additionally, co-printing of cell-seeded alginate scaffold confirmed that gene expression of both chondrocyte and adipocyte were remarkably enhanced compared to that of the control group. Mannoor et al. (2013) were able to integrate 3D biological tissue and electronic components. Alginate hydrogel with chondrocyte and silver nanoparticles was printed to structurally mimic human ears. Then, cochlea-shaped electrodes for hearing were inserted into the hydrogel construct, which was referred to as “cyborg ears.” Cell viability of the cyborg ear was 91.3 ± 3.9%, which is an adequate biocompatibility for the application.

Recently, an inorganic–organic hybrid of silica-poly(tetrahydrofuran)/PCL was 3D printed to fabricate scaffolds for articular cartilage regeneration (Tallia et al., 2018; Li et al., 2020). The silica network and organic component was forming co-networks via covalent bonding, which displayed elasticity, self-healing ability, and bioactivity. The scaffold with a grid-like structure mimicked the compressive behavior of cartilage, and *in vitro* chondrogenic differentiation was observed.

### Heart Valve

**FIGURE 3:**
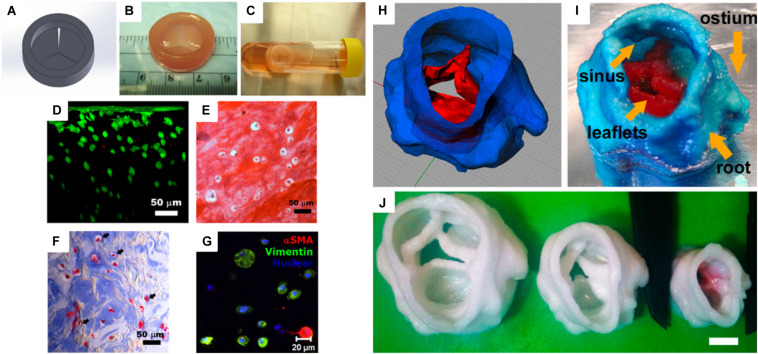
**(A)** Computer-aided design (CAD) model of a heart valve. **(B)** Bioprinted methacrylated hyaluronic acid/gelatin heart valve conduit. **(C)** The hydrogel hybrid conduit after 7 days of static culture, and **(D)** cross-sectional view of a live/dead cell viability assay. **(E)** Safranin-O staining image and **(F)** Masson’s Trichrome staining images showed that the heart valve conduit was composed of collagen type II and GAG. **(G)** Representative immunohistochemical staining image of αSMA, vimentin, and nuclei (Duan et al., 2014). **(H)** Porcine aortic valve model and **(I)** 3D printed scaffold with two types of PEG-DA inks [root: 700 molecular weight (MW) PEG-DA and leaflets: 700/8000 MW PEG-DA]. **(J)** Scaffolds were printed with 700 MW PEG-DA at different scales for fidelity analysis. The inner diameters (ID) were 22, 17, and 12 mm. Scale bar = 1 cm (Hockaday et al., 2012) (Reproduced with permission from Hockaday et al., 2012; Duan et al., 2014).

Heart is one of the essential organs in human physiology, which consists of various muscles to pump blood in the circulatory system. Heart valves, two in atria and the other two in ventricle chambers, are important for the blood circulation since they prevent backward flow (Dasi et al., 2009). In 2000, the American Heart Association announced that 87,000 replacement surgeries occurred (Flanagan and Pandit, 2003). Specifically, aortic valve disease is one of the serious cardiovascular diseases that are usually treated by replacement of the valves. Many researchers have studied artificial heart valves using various polymeric materials, such as PGA, PLA, collagen, and fibrin. Similar to the scaffolds described in the previous sections, inherent structures and mechanical properties are also important for designing heart valve conduits. Therefore, a 3D printing technique has been applied to this research field for several years (Hockaday et al., 2012). For heart valve engineering, hydrogels are promising materials due to their physicochemical and mechanical stability while they are hydrated. Furthermore, hydrogels are permeable for nutrients and waste transportation. Duan et al. (2014) fabricated human aortic valvular interstitial cell (HAVIC)-encapsulated heart valve conduits with photo-crosslinkable methacrylated hyaluronic acid (Me-HA) and methacrylated gelatin (Me-Gel) hydrogels. The viscosity of the hydrogel conduits was optimized and tuned by applying different hydrogel concentrations. The 3D bioprinted hydrogel conduits confirmed cell viability and remodeling potential for initial collagen and glycosaminoglycan matrix formation (Figures 3A–G). Hockaday et al. (2012) printed heart valve scaffolds with photo-crosslinkable poly(ethylene glycol)-diacrylate (PEG-DA). The scaffold was fabricated with two types of PEG-DA with different molecular weights in order to meet the heterogeneous mechanical properties of aortic valves. The conduit had high elastic modulus and nearly 100% cell viability (Figures 3H–J).

### Blood Vessel/Trachea

In the United States, coronary artery bypass grafting surgeries are performed more than 400,000 per year. Critical drawbacks of the surgeries are graft damages during harvesting procedure, poor long-term patency, and donor morbidity. Therefore, there are high demands for the development of artificial blood vessels that can overcome the current shortcomings. An ideal artificial blood vessel should be biocompatible, anti-thrombogenic, and durable, and have comparable compliance with structural density to that of the native blood vessels (Mosadegh et al., 2015). Wu et al. (2011) fabricated a biomimetic 3D microvascular network based on hydrogel matrix. The blood vessel’s branching pattern was designed by omnidirectional printing of sacrificial ink in a photo-crosslinkable hydrogel matrix. The authors suggested that this technique can be applied to 3D cell culture; however, there were no cell tests performed. Similar work was performed by Lee et al. (2014b); functional vascular channels with perfused open lumen were fabricated through 3D bioprinting of collagen matrix with liquefying fugitive ink. Gelatin with endothelial cell (EC) was used as a fugitive ink, which protected from plasma protein and dextran molecule. Additionally, human umbilical vein endothelial cells (HUVECs) were cultured in the vascular channel, which was successfully aligned along the flow direction. The gene expression analysis confirmed that the 3D printed vascular channels had high potential for tissue engineering application.

Fibrin is a natural polymer formed by polymerization of fibrinogen and thrombin. It is present in human blood and involved in the wound healing process. Cui and Boland (2009) 3D printed human microvascular endothelial cell (HMVEC)-seeded bioink of thrombin and Ca^2+^ solution into a fibrinogen substrate. The scaffold was composed of fibrin channels with aligned HMVECs, and 21 days of cell culture confirmed tubular structure formation inside the channels. Poly(propylene fumarate)-based aorta graft was synthesized via digital light stereolithography technique (Melchiorri et al., 2016). The biodegradable polymer was 3D printed to an MRI/CT scanned structure, which showed a possibility to design patient-specific aorta grafts. Additionally, the scaffold was able to confirm bioactive properties *in vivo* with comparable mechanical strength to that of human aorta.

**FIGURE 4:**
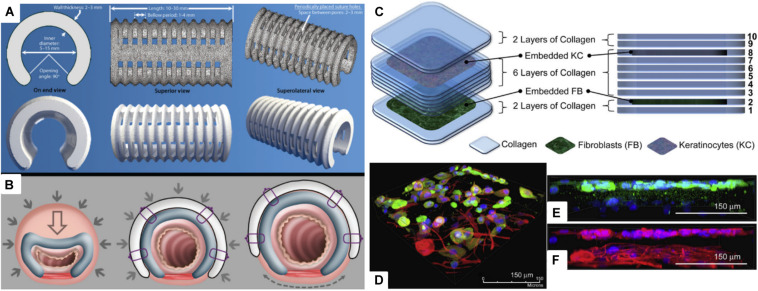
**(A)** Virtual rendering of tracheobronchial splint STL file in top, bottom, and side views. Inner diameter, length, thickness, and suture hole spacing were patient-specifically designed, and then it was placed over an airway through the 90° opening angle. **(B)** The mechanism of the tracheobronchial splint. Filled arrows signify intrathoracic pressure when breathing out, and empty arrows represent reducing vector values. Dashed arrow indicates the vector movement of a splint according to the airway growth (Morrison et al., 2015). **(C)** Representative scheme of a multi-layered collagen scaffold for tissue regeneration. Primary adult human dermal fibroblast-seeded collagen is printed in the 2nd layer, and primary adult human epidermal keratinocyte embedded collagen layer is deposited in the 8th layer. **(D)** Immunofluorescent image of the 3D printed multi-layered scaffold with fibroblast and keratinocyte on a tissue culture dish. **(E)** Keratinocyte layer with keratin, and **(F)** keratinocyte and fibroblast layer with β-tubulin (Lee et al., 2009) (Reproduced with permission from Lee et al., 2009; Morrison et al., 2015).

Tracheal structure restoration and scaffold fabrications are also in great demand. Forty-three percent of pediatric patients who went through tracheostomy experience respiratory arrest due to tube occlusion (Carr et al., 2001; Berry et al., 2010). However, if the pediatric patients are supported by a temporary scaffold for 24 to 36 months, the airway growth can naturally resolve the disease. Morrison et al. (2015) were able to produce a personalized and biodegradable tracheal splint through the SLS technique. PCL powders were mixed with hydroxyapatite, which was used as a flowing agent for the laser sintering process. PCL splints were successfully implanted to pediatric patients, and they were able to expand over time with airway growth (Figures 4A,B). Chang et al. (2014) 3D printed PCL scaffolds through an FDM-type printer for tracheal regeneration. The scaffold was coated with MSC-seeded fibrin to enhance bioactivity. *In vivo* study confirmed that the scaffolds were mechanically stable and able to reconstruct trachea within 8 weeks of implantation. Kim et al. (2018) produced a ring-like cartilaginous trachea scaffold through 3D bioprinting with digital light processing technique. Chondrocytes were encapsulated in methacrylated silk fibroin, which made cross-linking possible through UV light exposure. This cell-loaded hydrogel scaffold showed homogeneously distributed cells and cartilage tissue formation *in vitro*. The chemically modified silk fibroin ink was also printed to heart, lung, and vascular shapes, which confirmed that the bioink and printing method can be applied to various tissue engineering applications.

### Liver

The liver is a fundamental organ that is responsible for multifunctional metabolic activities. Although liver transplant has been practiced for a long time, the procedure is costly, patient survival rate is poor, and there is a shortage of organ donors (Wang et al., 2007). Wang et al. (2006) made a 3D construct of gelatin hydrogel with hepatocyte as an ECM and 2.5% glutaraldehyde as a cross-linking agent. They were able to confirm that hepatocytes in gelatin construct can survive over 2 months in *in vitro* cell culture and retain their 3D structure for a month. A chitosan–gelatin hybrid scaffold was developed to biomimic the architecture of natural liver. A highly porous and well-organized structure was fabricated by a combination of 3D printing, micro-replication, and freeze-drying techniques. The novel scaffold was also composed of intrinsic fluidic channels and hepatic chambers. Firstly, a resin mold was fabricated by the SLA technique to cast polydimethylsiloxane (PDMS) for creating a micro-replication mold. Chitosan–gelatin solution was cast in the PDMS mold followed by freeze-drying to produce a porous structure. Biodegradability and hepatocyte growth were confirmed through 7-day cell culture. More importantly, albumin secretion and urea synthesis were evident, which are representative evaluation for hepatocyte functionality (Jiankang et al., 2009). Lee et al. (2016) 3D printed PCL to a grid-like structure as a primary framework to mechanically support collagen bioinks. HUVECs, human lung fibroblasts (HLFs), and hepatocytes were encapsulated in collagen inks, printed in between PCL struts for angiogenesis. The authors also evaluated albumin secretion and urea synthesis for confirming hepatocytes functionality and angiogenesis. Specifically, hepatocyte-, HLF-, and HUVEC-containing constructs showed highest albumin secretion and urea formation compared to that of hepatocytes only and hepatocyte- and HLF-containing groups.

### Skin

Skin is the largest organ in our body that is responsible for various functions, such as preventing loss of body fluid, acting as a barrier against pathogenic bacterium and thermotaxis, and regulating body temperature (Bonvallet et al., 2015; Ojeh et al., 2015). Severe acute and chronic wounds (i.e., burns, diabetic ulcer, pressure sores, and lesion) effect loss of dermal tissues. Skin grafts have limitations in antigenicity and shortage of transplantable tissues; therefore, there are high demands for skin regeneration (Tchemtchoua et al., 2011; Wang et al., 2013). Lee et al. (2009) used a 3D bioprinter with four-channel dispensers to print stratified skin layers. PDMS substrate was first coated with sodium bicarbonate, a pH-altering cross-linking agent. Then, collagen was printed layer by layer to fabricate a multi-layered skin construct. Specifically, among the 10 layers of skin construct, the 2nd and 8th collagen layers were embedded with cells, fibroblast and keratinocyte, respectively. Several advantages of this fabrication method include the following: the scaffold can be made on irregular surfaces, as long as cross-linking agent coating is possible, and other types of hydrogels can substitute collagen, if they are cross-linkable. Additionally, this was the first study to 3D print both keratinocyte and fibroblast for skin regeneration (Figures 4C–F). Koch et al. (2010, 2012) also 3D printed keratinocyte- and fibroblast-embedded collagen for skin tissue engineering. Laser-assisted bioprinting (LaBP) with laser-induced forward transfer technique was used to produce 3D scaffolds. The LaBP method is advantageous over other bioprinting techniques since higher-resolution cell printing is possible with greater cell density. Also, various hydrogels can be printed regardless of their viscosity. The skin scaffold, which was printed in micro-scale, confirmed that each cell layer did not blend into each other. Ten days of culture confirmed the cell vitality of the cells embedded in the scaffold, and collagen layers did not intermix with each other.

## Conclusion and Perspectives

Three-dimensional printing is one of the most promising technologies in tissue engineering and regenerative medicine to fabricate advanced 3D scaffolds. It allows to produce more defined and biomimetic scaffolds with bioactive factors to enhance their functionalities. Although there have been numerous studies in 3D printing for biomedical applications, there are still much room for improvement (Jakus et al., 2016; Zhu et al., 2016). Optimization of printable inks, standardization of printing methods, and higher reproducibility with mass production are major challenges. 4D printing, an integration of 3D printing with time, has emerged recently. This technology allows the printed materials to change their physical forms or functionalities when excited by an external stimulus, such as temperature, water, magnetism, and pH (Gao et al., 2016; Gladman et al., 2016). Since human body is a complex environment with various stimuli, 4D printing technology is receiving a lot of attention for medical implant surgeries. 3D bioprinting of stem cells has shown unprecedented possibilities for producing tissue constructs from bone to skin. As bioprinting technology advances, printing induced pluripotent stem (iPS) cells could take current bioactive scaffolds a step closer to regenerate patient-specific tissues and organs. iPS cells are known to have advantages over embryonic stem cells since they can be derived from patients for autologous cell treatment. However, the reprogramming process is not fully understood so far (Yamanaka, 2009). 3D bioprinting is an encouraging technology for future regenerative medicine. It allows to deliver, or mount, cells and physicochemical factors that are essential for tissue regeneration. Furthermore, patient-specific therapies are one of the essential technologies for hospital factory, where damaged organ rendering is produced by medical imaging, and defect regenerating construct is printed with patient’s cells, plasma, and tissues in the operating theater. The authors are confident that the progress in 3D printing technology will foster and enhance personalized regenerative medicine.

## Author Contributions

JC and HI drafted the initial manuscript with guidance by YJ. SK, YJ, and JP made contributions and modifications according to their field of expertise. All authors reviewed and approved the manuscript.

## Conflict of Interest

The authors declare that the research was conducted in the absence of any commercial or financial relationships that could be construed as a potential conflict of interest.
